# Proportion and risk factors for death by euthanasia in dogs in the UK

**DOI:** 10.1038/s41598-021-88342-0

**Published:** 2021-05-04

**Authors:** Camilla Pegram, Carol Gray, Rowena M. A. Packer, Ysabelle Richards, David B. Church, Dave C. Brodbelt, Dan G. O’Neill

**Affiliations:** 1 Pathobiology and Population Sciences, The Royal Veterinary College, Hawkshead Lane, North Mymms, Hatfield, AL9 7TA Herts UK; 2 School of Law and Social Justice, University of Liverpool, Chatham St., Liverpool, L69 7ZR UK; 3 Clinical Sciences and Services, The Royal Veterinary College, Hawkshead Lane, North Mymms, Hatfield, AL9 7TA Herts UK

**Keywords:** Risk factors, Epidemiology

## Abstract

The loss of a pet can be particularly distressing for owners, whether the method of death is euthanasia or is unassisted. Using primary-care clinical data, this study aimed to report the demographic and clinical factors associated with euthanasia, relative to unassisted death, in dogs. Method of death (euthanasia or unassisted) and clinical cause of death were extracted from a random sample of 29,865 dogs within the VetCompass Programme from a sampling frame of 905,544 dogs under UK veterinary care in 2016. Multivariable logistic regression modelling was used to evaluate associations between risk factors and method of death. Of the confirmed deaths, 26,676 (89.3%) were euthanased and 2,487 (8.3%) died unassisted. After accounting for confounding factors, 6 grouped-level disorders had higher odds in euthanased dogs (than dogs that died unassisted), using neoplasia as the baseline. The disorders with greatest odds included: poor quality of life (OR 16.28), undesirable behaviour (OR 11.36) and spinal cord disorder (OR 6.00). Breed, larger bodyweight and increasing age were additional risk factors for euthanasia. The results highlight that a large majority of owners will face euthanasia decisions and these findings can support veterinarians and owners to better prepare for such an eventuality.

## Introduction

Dogs are the most popular mammalian species kept as companion animals globally, largely stemming from the deep human–dog bond^1^. Positive human–dog relationships can lead to physiological and emotional changes that benefit both the human owners as well as their dogs^1^. However, given overall median longevity in dogs is reported as 12.0 years^2^, long-term dog ownership means that many owners are likely to face losing several dogs during a typical human lifetime. Whether the method of death is euthanasia or is unassisted, the loss of a pet can be particularly distressing for owners^3^. However, societal attitudes to pet death are changing. Although sometimes regarded as “disenfranchised grief”^4,5^, the death of a cherished pet is now acknowledged as a significant life event for owners, with some employers even granting compassionate leave for those affected^6^. End-of-life care for pets has emerged as a growing discipline within veterinary medicine, with the pet hospice movement expanding^7^. Designed to support clients and animal patients through difficult decisions and care pathways, the veterinarian’s role in pet hospice care is often non-clinical, with help-oriented roles such as educator, supporter, guider and facilitator overshadowing the role as veterinary medical expert^8^. Euthanasia in veterinary medicine is taken to mean a painless death following a standard euthanasia protocol^9^ although the reasons behind the decision to euthanase an animal are manifold. In contrast, the suggested definition for euthanasia for human patients is a death that is intended to relieve the patient’s suffering^10^.

The wider process of euthanasia has been described by veterinarians as representing “the best and the worst” of the profession^11,12^, with 86.4% of deaths in dogs presenting to veterinary clinics recorded as involving euthanasia in the UK^2^. Dealing with animal death, and especially euthanasia, is a major challenge for veterinary professionals^13^ that can be morally complex and stressful as well as negatively impactful on mental health^14^. Veterinarians are often pushed to balance the interests of a chronically sick or unwanted animal with those of the client, who may insist that treatment should be continued or discontinued against veterinary advice. Veterinarians may be reluctant to offer euthanasia as an option, viewing it as an admission of failure of their treatment skills^15^, or because the animal may be suffering from a treatable condition but the client may be unable to afford the proposed treatment due to lack of pet insurance, a situation termed ‘economic euthanasia’^16^. In the context of undesirable behaviours, some owners may be unwilling or unable to invest the necessary time to treat these behaviour issues. Despite such ethically problematic situations, many veterinarians place great importance on their roles as care-givers in the context of pet death^11,13^.

Much of the previous literature has focused on shelter dogs, particularly in the US, comparing euthanased dogs to non-euthanased dogs to evaluate risk factors^17–20^, limiting the generalisability to the UK primary-care population. One study on US shelter dogs reported that crossbred dogs had 1.8 times the risk for euthanasia (relative to non-death) compared with purebred dogs. Additionally, the risk of euthanasia increased with age for crossbred dogs, but not for purebred dogs^17^. A UK study based on owner questionnaires of 3126 dogs that died reported that the method of death was euthanasia in 52.0% of dogs, and that disease (29.3%), old age (20.7%) and behavioural problems (2.0%) were the most common reasons for euthanasia. The most common reasons for death in the remaining 47.9% unassisted deaths were illness (35.3%), natural causes (7.7%), road traffic accidents (3.2%) and other accidents (1.7%)^21^. The epidemiology of dog euthanasia in Canada has been reported, based on veterinarians’ overall perceptions of euthanasia cases rather than based on the clinical notes, with “old age” stated as the reason for euthanasia by practicing veterinarians in 39.8% of euthanased dogs. “Old age” was followed by terminal sickness (30.2%), aggression (9.0%) and other behavioural abnormalities (4.2%)^22^. Reported diseases of old age in human healthcare include sensory deterioration, chronic conditions such as cardiovascular disease and osteoarthritis, and cognitive ageing^23^, and similar conditions are commonly reported in ageing dogs^24^. A Brazilian study based on a university teaching hospital population of dogs reported infectious or parasitic diseases, diseases caused by physical agents and neoplasia as the main reasons for euthanasia and causes of unassisted death collectively^25^. Whilst some of these previous studies have touched on differences in causes of death in dogs that were euthanased compared with unassisted deaths, the causes of death have been grouped into relatively broad categories and this has not been the primary research focus. Additionally, there is a lack of more recent research in to canine euthanasia, particularly UK primary-care based, highlighting the need for up-to-date information.

The most common causes of death in dogs under primary veterinary care in the UK have been reported as neoplastic diseases, musculoskeletal disorders and neurological disorders^2^. When broken down by age, the most common causes of death in dogs before 3 years old were behavioural abnormality, gastrointestinal disorder and road traffic accident, whilst in dogs over 3 years neoplastic diseases, musculoskeletal disorders and neurological disorders were the most common causes^2^. These findings are similar to other age-specific reports, in which neoplastic, cardiovascular, musculoskeletal, respiratory and neuromuscular disorders were common causes of death in older dogs, whilst infectious, parasitic, gastrointestinal and traumatic diseases were common causes of death in younger dogs^26–28^. However, these studies focused on mortality as a whole, rather than sub-categorising dogs by their method of death as euthanased or unassisted.

Using anonymised veterinary clinical data from the VetCompass Programme^29^, this study aimed to explore the method of death in dogs. The specific objectives were to report proportional euthanasia or unassisted death, as well as the demographic and clinical factors associated with euthanasia (relative to unassisted death), in dogs under primary veterinary care in the UK during 2016. By comparing euthanasia to unassisted deaths, a range of causes of death could be assessed simultaneously to evaluate their relative impacts on decision-making to opt for euthanasia compared with an unassisted death. This comparison also allows identification of the factors that negatively influence owner-interpreted canine quality of life but are less amenable to palliative treatment, thus leading to decisions for euthanasia. An additional objective was to report method of body disposal in euthanased dogs compared with dogs that died unassisted. Given some suggestive previous evidence that “old age” is commonly cited as a reason for euthanasia^21,22^, the study hypothesised that odds of euthanasia relative to unassisted deaths in dogs would rise with aging. Due to differences in causes of death according to age, this may in turn result in differences in the method of death, although to the best of the authors’ knowledge this has not been previously investigated. Improved understanding of the influence of demographic and clinical factors promoting euthanasia in dogs, relative to unassisted death, could aid veterinarians in end of dog-life discussions with owners and thus improve canine health management. Clients look to veterinarians for medical and non-medical advice in “stressful and complex” situations such as end-of-life care^15^. Therefore, end-of-life conversations may include discussion about whether euthanasia or natural death might be more likely and indeed, which may be preferable, depending on the patient’s condition^8^. By providing benchmark data for the relative proportion of deaths that involve euthanasia, and for the relative impact from demographics and disorders on euthanasia decision-making, owners and veterinary professionals may find it easier to discuss end-of-life options, to reach a final decision and to be comfortable with these decisions based on a feeling of broader support from the reported actions of others in similar situations. In addition, the study findings could act as hypothesis generators that can ultimately deepen our understanding of when, why and how dogs commonly die in the UK. This information can help to direct reforms aimed at improving welfare, such as the management of specific disorders or even breed selection. Additionally, it may provide basic guidance on which disorders are appropriate for consideration of palliative treatment and which may require a more rapid decision for euthanasia.

## Results

### Demography and descriptive statistics

The study population included 905,544 dogs from 626 clinics in the VetCompass database under veterinary care in the UK during 2016. The analysis included a random sample of 29,865 confirmed deaths in dogs after January 1^st^, 2016 in the available records. Of the deaths, 26,676 (89.3%) were euthanased, 2,487 (8.3%) died unassisted, whilst the method in 702 (2.4%) were unrecorded and excluded from further analysis, leaving 29,163 dogs in the analysis. Of the dogs with information on the method of death recorded, 91.5% (95% CI 91.1 to 91.8) were euthanased and 8.5% (95% CI 8.2 to 8.9) died unassisted. Data completeness were: breed 99.6%, age 98.7%, sex-neuter status 99.7%, insurance status 100.0% and bodyweight 62.6%.

Descriptive statistics included 26,676 euthanased dogs and 2487 dogs that died unassisted (Table 1). The median age at death of euthanased dogs (12.1 years, IQR 9.6–14.1, range 0.0–21.9) was older than the median age of dogs that died unassisted (9.9 years, IQR 6.4–12.5, range 0.0–21.0) (p < 0.001). The median bodyweight of euthanased dogs (18.4 kg, IQR 9.7–28.9, range 0.2–94.0) was heavier than the median bodyweight of dogs that died unassisted (15.0 kg, IQR 8.4–28.0, range 0.2–92.0) (p < 0.001). Cause of death was not recorded in 3668/26,676 (13.8%) euthanased dogs and 1213/2487 (48.8%) dogs that died unassisted. The most commonly recorded causes of death (grouped-disorders) amongst euthanased dogs were neoplasia (2658; 11.6%), collapsed (2558; 11.1%), mass (1843; 8.0%) and behaviour disorder (1679; 7.1%). The most commonly recorded causes amongst dogs that died unassisted were heart disease (251; 19.7%), traumatic injury (217; 17.0%), collapsed (100; 7.8%) and lower respiratory tract disorder (97; 7.6%).

**Table 1:** Demography and causes of death for euthanased dogs (n = 26,676) and dogs that died unassisted (n = 2487) attending primary-care veterinary practices in the VetCompass Programme in the UK during 2016.

Variable	Category	Euthanasia count (%)	Unassisted death count (%)
Breed	Crossbreed	6053 (22.7)	458 (18.4)
	Purebred—other	6839 (25.6)	721 (29.0)
	Labrador Retriever	2315 (8.7)	145 (5.8)
	Staffordshire Bull Terrier	2077 (7.8)	134 (5.4)
	Jack Russell Terrier	1463 (5.5)	119 (4.8)
	German Shepherd Dog	989 (3.7)	115 (4.6)
	Yorkshire Terrier	919 (3.4)	116 (4.7)
	West Highland White Terrier	897 (3.4)	98 (3.9)
	Border Collie	855 (3.2)	69 (2.8)
	Cocker Spaniel	850 (3.2)	62 (2.5)
	Cavalier King Charles Spaniel	703 (2.6)	130 (5.2)
	Boxer	587 (2.2)	45 (1.8)
	Shih-tzu	534 (2.0)	52 (2.1)
	Golden Retriever	466 (1.7)	27 (1.1)
	Rottweiler	472 (1.8)	28 (1.1)
	Bulldog	208 (0.8)	78 (3.1)
	Pug	151 (0.6)	41 (1.6)
	Chihuahua	200 (0.7)	34 (1.4)
	Not recorded	98 (0.4)	15 (0.6)
Bodyweight (kg)	< 10	4397 (16.5)	535 (21.5)
	10 to < 20	4570 (17.1)	418 (16.8)
	20 to < 30	3878 (14.5)	290 (11.7)
	≥ 30	3833 (14.4)	340 (13.7)
	Not recorded	9998 (37.5)	904 (36.3)
Age at death (years)	< 6	2387 (9.7)	557 (22.4)
	6 to < 9	3113 (12.6)	473 (19.0)
	9 to < 12	7277 (27.3)	698 (28.0)
	12 to < 15	9503 (38.5)	549 (22.1)
	≥ 15	4076 (16.5)	165 (6.6)
	Not recorded	320 (1.2)	45 (1.8)
Sex-Neuter status	Female entire	4887 (18.3)	532 (21.4)
	Female neutered	7799 (29.2)	667 (26.8)
	Male entire	6369 (23.9)	681 (27.4)
	Male neutered	7544 (28.3)	602 (24.2)
	Not recorded	77 (0.3)	5 (0.2)
Insurance	Non-insured	22,209 (83.3)	2028 (81.5)
	Insured	4467 (16.7)	459 (18.5)
Vet Group	1	11,442 (42.9)	1058 (42.5)
	2	7556 (28.3)	843 (33.9)
	3	1813 (6.8)	95 (3.8)
	4	5737 (21.5)	467 (18.8)
	5	128 (0.5)	24 (1.0)
Grouped-level disorder	Neoplasia	2658 (11.6)	86 (6.8)
	Collapsed	2558 (11.1)	100 (7.8)
	Mass	1843 (8.0)	23 (1.8)
	Behaviour disorder	1679 (7.3)	7 (0.5)
	Brain disorder	1634 (7.1)	96 (7.5)
	Musculoskeletal disorder	1356 (5.9)	20 (1.6)
	Poor quality of life	1301 (5.7)	2 (0.2)
	Heart disease	1047 (4.6)	251 (19.7)
	Enteropathy	853 (3.7)	66 (5.2)
	Lower respiratory tract disorder	850 (3.7)	97 (7.6)
	Spinal cord disorder	815 (3.5)	4 (0.3)
	Kidney disease	771 (3.4)	19 (1.5)
	Inappetence	518 (2.3)	6 (0.5)
	Endocrine system disorder	446 (1.9)	30 (2.4)
	Lethargy	372 (1.6)	12 (0.9)
	Haematopoietic disorder	335 (1.5)	26 (2.0)
	Upper respiratory tract disorder	308 (1.3)	34 (2.7)
	Traumatic injury	260 (1.1)	217 (17.0)
	Foreign body	59 (0.3)	16 (1.3)
	Complication associated with clinical care	52 (0.2)	27 (2.1)
	Other	3293 (14.3)	135 (10.6)

### Method of managing the remains

**Figure 1:**
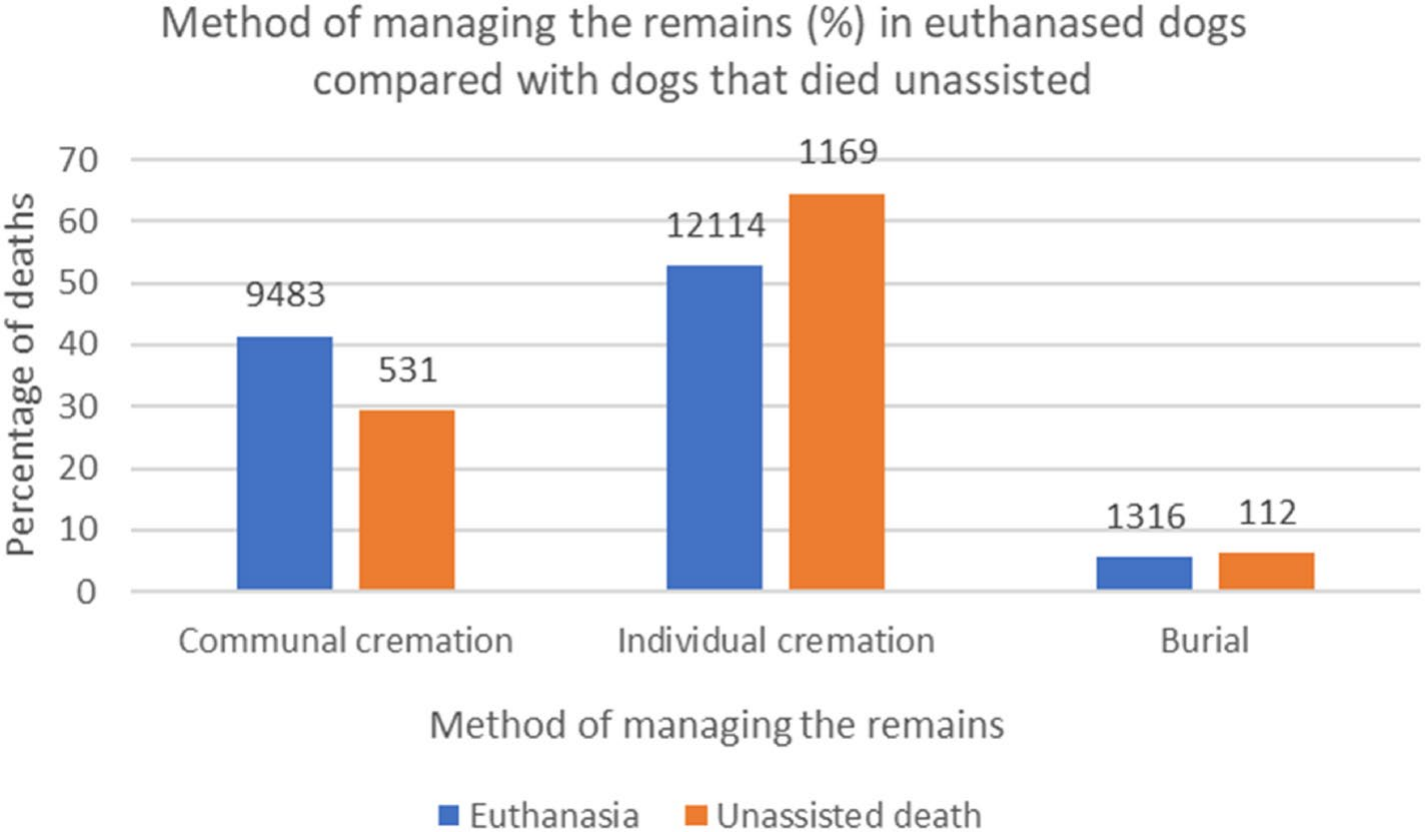
Method of managing the remains (%) of euthanased dogs (n = 22,913) and dogs that died unassisted (n = 1812) attending primary-care veterinary practices in the VetCompass Programme in the UK during 2016. The count of dogs in each category is given at the top of each bar.

Of the euthanased dogs, the method of managing the remains was not recorded in 3740 (14.0%) and the remains of 23 (0.09%) dogs were managed by means other than cremation or burial, such as those donated to science or used for taxidermy. Of the dogs that died unassisted, the method of managing the remains was not recorded in 674 (27.1%) dogs and the remains of 1 dog (0.04%) were managed by means other than cremation or burial. The aforementioned dogs were excluded from further analysis. Based on a chi-squared test, there was a significant difference between the proportions of dogs buried, individually cremated or communally cremated between dog that were euthanased and dogs that died unassisted (p < 0.001). (Fig. 1).

### Risk factors for euthanasia compared with unassisted death

Univariable logistic regression modelling for risk factors for euthanasia compared with unassisted death in dogs identified seven variables that were liberally significant and were carried forward for multivariable modelling: Breed, Bodyweight (kg), Age at death (years), Sex-Neuter status, Insurance status, Vet Group and Grouped-level disorder. The final multivariable model retained five variables: Breed, Bodyweight (kg), Age at death (years), Vet Group and Grouped-level disorder (Table 2).

**Table 2:** Final multivariable model for risk factors associated with euthanasia in dogs under primary veterinary care in the UK during 2016 (n = 29,163). *Confidence interval.

Variable	Category	Odds Ratio	95% CI*	Category P-value	Variable P-value
Breed	Labrador Retriever	Base			< 0.001
	Rottweiler	1.76	1.12 to 2.76	0.014	
	Chihuahua	1.06	0.66 to 1.71	0.809	
	Boxer	1.03	0.71 to 1.49	0.888	
	Cocker Spaniel	0.99	0.70 to 1.40	0.964	
	Shih-tzu	0.94	0.64 to 1.38	0.760	
	Staffordshire Bull Terrier	0.94	0.72 to 1.23	0.643	
	Crossbreed	0.93	0.75 to 1.15	0.512	
	Golden Retriever	0.90	0.57 to 1.40	0.627	
	Jack Russell Terrier	0.82	0.61 to 1.10	0.184	
	Purebred—Other	0.81	0.66 to 0.99	0.044	
	German Shepherd Dog	0.76	0.58 to 1.01	0.059	
	Cavalier King Charles Spaniel	0.73	0.54 to 0.98	0.034	
	Border Collie	0.63	0.45 to 0.87	0.005	
	Yorkshire Terrier	0.63	0.46 to 0.85	0.002	
	West Highland White Terrier	0.53	0.39 to 0.72	< 0.001	
	Pug	0.47	0.30 to 0.75	0.001	
	Bulldog	0.32	0.23 to 0.47	< 0.001	
Bodyweight (kg)	< 10	Base			< 0.001
	10 to < 20	1.05	0.90 to 1.24	0.515	
	20 to < 30	1.24	1.03 to 1.49	0.024	
	≥ 30	1.18	0.98 to 1.42	0.084	
	Not recorded	1.36	1.18 to 1.57	< 0.001	
Age at death (years)	< 6	Base			< 0.001
	6 to < 9	1.38	1.18 to 1.62	< 0.001	
	9 to < 12	2.16	1.87 to 2.50	< 0.001	
	12 to < 15	3.82	3.28 to 4.44	< 0.001	
	≥ 15	5.92	4.82 to 7.26	< 0.001	
Vet Group	1	Base			< 0.001
	2	0.75	0.67 to 0.83	< 0.001	
	3	1.63	1.28 to 2.08	< 0.001	
	4	1.23	1.08 to 1.39	0.002	
	5	0.45	0.28 to 0.74	0.002	
Grouped-level disorder	Neoplasia	Base			< 0.001
	Poor quality of life	16.28	3.99 to 66.34	< 0.001	
	Undesirable behaviour	11.36	4.93 to 26.16	< 0.001	
	Spinal cord disorder	6.00	2.19 to 16.45	< 0.001	
	Inappetence	2.67	1.07 to 6.62	0.035	
	Mass	2.13	1.34 to 3.40	0.001	
	Musculoskeletal disorder	1.72	1.04 to 2.85	0.035	
	Kidney disease	1.29	0.77 to 2.16	0.341	
	Lethargy	0.80	0.43 to 1.49	0.490	
	Other	0.76	0.57 to 1.01	0.056	
	Collapsed	0.59	0.44 to 0.80	< 0.001	
	Brain disorder	0.53	0.39 to 0.72	< 0.001	
	Endocrine system disorder	0.46	0.30 to 0.70	< 0.001	
	Enteropathy	0.45	0.32 to 0.64	< 0.001	
	Haematopoietic disorder	0.44	0.28 to 0.69	< 0.001	
	Lower respiratory tract disorder	0.28	0.21 to 0.38	< 0.001	
	Upper respiratory tract disorder	0.26	0.17 to 0.40	< 0.001	
	Foreign body	0.18	0.10 to 0.34	< 0.001	
	Heart disease	0.14	0.10 to 0.18	< 0.001	
	Complication associated with clinical care	0.08	0.05 to 0.14	< 0.001	
	Not recorded	0.08	0.06 to 0.10	< 0.001	
	Traumatic injury	0.05	0.04 to 0.07	< 0.001	

After accounting for confounding using multivariable methods, there were 6/21 (28.6%) grouped-level disorders with higher odds in euthanased dogs than dogs that died unassisted, using neoplasia as the baseline. The disorders with greatest odds of euthanasia compared with unassisted death included: poor quality of life (odds ratio [OR] 16.28; 95% CI 3.99 to 66.34; p < 0.001), undesirable behaviour (OR 11.36; 95% CI 4.93 to 26.16; p < 0.001), spinal cord disorder (OR 6.00; 95% CI 2.19 to 16.45; p < 0.001) and inappetence (OR 2.67; 1.07 to 6.62; p = 0.035). Conversely, there were 12/21 (57.1%) disorders with lower odds of euthanasia compared with unassisted death, using neoplasia as the baseline. The disorders with lowest odds of euthanasia included: traumatic injury (OR 0.05; 95% CI 0.04 to 0.07; p < 0.001), disorder not recorded (OR 0.08; 95% CI 0.06 to 0.10; p < 0.001), complication associated with clinical care (OR 0.08; 95% CI 0.05 to 0.14; p < 0.001) and heart disease (OR 0.14; 95% CI 0.10 to 0.18; p < 0.001).

One breed showed increased odds of euthanasia compared with Labrador Retrievers: Rottweiler (OR 1.76; 95% CI 1.12 to 2.76; p = 0.014). Seven breeds showed reduced odds of euthanasia (in other words, higher odds of unassisted death) compared with Labrador Retrievers. The breeds with lowest odds of euthanasia included: Bulldog (OR 0.32; 95% CI 0.23 to 0.47; p < 0.001), Pug (OR 0.47; 95% CI 0.30 to 0.75; p = 0.001), West Highland White Terrier (OR 0.53; 95% CI 0.39 to 0.72; p < 0.001) and Yorkshire Terrier (OR 0.63; 95% CI 0.46 to 0.85; p = 0.002). Increasing age at death (years) was associated with increased risk of euthanasia relative to unassisted deaths, with dogs aged ≥ 15 years showing 5.92 times the odds of euthanasia (95% CI 4.82 to 7.26; p < 0.001) compared with dogs aged < 6 years. Bodyweight (kg) was associated with euthanasia, with dogs weighing 20 to < 30 kg at 1.24 times the odds of euthanasia (95% CI 1.03 to 1.49; p = 0.024) compared with dogs < 10 kg. The Hosmer–Lemeshow test indicated acceptable model fit (p = 0.499) and the area under ROC curve (0.848) indicated good predictive ability.

## Discussion

This is the first study to report proportional death rates for dogs by euthanasia versus unassisted in the population of animals under primary veterinary care in the UK. The study additionally reports on demographic and clinical factors associated with euthanasia relative to unassisted death. Much of the previous literature focused on risk factors for euthanasia in animals in shelters, particularly in the US, limiting the generalisability to the UK dog population under primary care^17–20^. Instead, the current study included deaths in 29,163 dogs under primary care with the aim of getting a deeper understanding of the processes around the deaths of dogs in the wider owned dog population. The results showed that the majority of deaths in dogs involved euthanasia, with 26,676 (91.5%) dogs euthanased compared with 2487 (8.5%) that died unassisted. It therefore provides novel insights in to the demographic and clinical factors associated with euthanasia relative to unassisted deaths, providing an evidence-base for veterinarians on the aspects influencing euthanasia and unassisted deaths in dogs. In turn, this will enable veterinarians to use benchmark data collected from this broad range of UK veterinary practice to support owners with an evidence-base to assist their decision-making process.

The results support the study hypothesis that the odds of euthanasia relative to unassisted deaths in dogs rises with aging. Increasing age was associated with an increased risk of euthanasia, with dogs aged ≥ 15 years at 5.92 times the odds of euthanasia, relative to an unassisted death, compared with those < 6 years. Previous studies have cited “old age” as a major reason for euthanasia^21,22,30^, although to the authors’ knowledge, this risk relative to unassisted death has not been quantified previously. Given that age is the greatest risk factor not only for the probability of death, but also for the majority of morbidities associated with mortality^31–34^, the increased risk of euthanasia relative to unassisted death identified in aging dogs in the current study is not unsurprising. However, this finding does highlight the increasing importance of quality of life (QOL) and euthanasia decision-making discussions between veterinarians and owners as dogs age. Decision-making in older animals may be more likely to include euthanasia as an option, as has been reported in a previous study based on dogs and cats diagnosed with diabetes mellitus^35^. Conversely, veterinarians may be more reluctant to agree to euthanasia for younger, treatable patients^14^. This reflects the current study findings, given younger dogs were at greater risk of an unassisted death compared with euthanasia. Although euthanasia may be used to relieve animals from suffering, their health and welfare prior to this decision should still be considered and suffering minimised. It is of concern that in a previous study, 69% of dogs aged over 10 did not receive veterinary care during the 18 months leading up to euthanasia^36^. Promoting the welfare of geriatric patients is of high priority, including palliative veterinary care where appropriate^37^.

Disorders with greatest probability of euthanasia compared with an unassisted death (using neoplasia as the baseline) included: poor quality of life, undesirable behaviour and spinal cord disorder. Improved nutrition and healthcare have contributed to extended canine lifespans, therefore maintaining QOL is a growing concern in companion animal practice^38^. QOL in pets has been defined as states of comfort or discomfort representing a combination of physical and non-physical factors^39,40^. However, QOL is often assessed by owners and is somewhat subjective^38^. More recently, QOL tools have been developed to help optimize and standardize euthanasia decision-making in pets^38,41^. Given that poor QOL was identified as a strong risk factor for euthanasia (OR 16.28), this suggests that veterinary professionals and owners are considering QOL as a determining factor when deciding on euthanasia. Although, the current study did not extract information on proportional usage of validated QOL tools in these considerations. This could be a useful area of future research. The use of validated QOL tools could assist veterinary professionals and owners to better recognise and manage functional decline as canine patients age^38^. Additionally, guidelines produced by the animal hospice movement^42^ may prove useful in assessing QOL and, in turn, predicting quality of death^8^. The magnitude of the odds ratio for poor QOL is not surprising, given poor quality of life is associated with discomfort^39,40^, and therefore veterinarians and owners may be more likely to opt for euthanasia rather than allow the dog to die unassisted.

The current study identified undesirable behaviour as a significant risk factor for euthanasia relative to unassisted death (OR 11.36). Behavioural problems are considered an important factor in the euthanasia of dogs^2,43,44^, with previous reports suggesting behavioural problems account for 2–39% of canine euthanasia in veterinary practices^21,45,46^ and 50–70% in animal shelters^43,47^. The current study identified undesirable behaviour as the fourth most common reason for euthanasia, accounting for 7.3% of euthanasia deaths. Both genetic and environmental factors influence behavioural development and there is growing evidence to suggest that educating owners about puppy-raising practices, and the provision of ongoing socialisation and habituation, can reduce the incidence of problem behaviour^48^. Steps to reduce the incidence of problem behaviours may not only improve the welfare of individual dogs (if problems are associated negative emotional states and/or inappropriate punishment from caregivers), but may also reduce the number of dogs euthanased due to behavioural issues. It might therefore be apt for veterinary professionals to prioritise behavioural discussions at initial puppy consultations. Veterinary practitioners often lack confidence in dealing with behavioural problems, which has been attributed to poor coverage of the subject in veterinary education^49^. The current study findings support the suggestion for improved provision of behaviour medicine in veterinary education^49^. It should be noted that very few dogs with an undesirable behaviour will die naturally, reflected in the finding that only 7 dogs in the current study died unassisted of an undesirable behaviour compared with 1679 dogs that were euthanased. Therefore, absolute values as well as the relative odds ratio should be considered when interpreting these results. Additionally, behaviours may be labelled as undesirable by the owners, and what is considered undesirable for one owner might be acceptable for another^44^. Therefore, owner perception of the desirability of any specific behaviour may affect the likelihood for euthanasia.

Spinal cord disorder was identified as a significant risk factor for euthanasia relative to unassisted death in the current study (OR 6.00). Disorders of the spinal cord include congenital defects, degenerative diseases, inflammatory and infectious diseases, tumours, injury and trauma and vascular diseases^50^. Typical age at onset and presentation vary according to aetiology, but diagnosis of spinal cord disorders often involves advanced imaging such as computed tomography (CT) or magnetic resonance imaging (MRI)^50^. CT and MRI scanning are complex and expensive procedures^51^, therefore may not be accessible to all owners. If advanced imaging and possible subsequent surgery are required to treat a spinal cord disorder, it might be that an owner opts for euthanasia if the diagnostic and treatment options are not financially viable and there is not a realistic alternative to protect their dog’s QOL. Such data were not available in the current study, but suggest an important area for future research.

The disorders with lowest odds of euthanasia (and therefore the highest odds of unassisted death) were traumatic injury, disorder not recorded, complication associated with clinical care and heart disease. Traumatic injuries are a common emergency presentation^52^, with a US study reporting prevalence of traumatic injury at two large university veterinary hospitals as approximately 13%^53^. The most common causes of trauma in the US study were motor vehicle accident (53.2%), unknown cause (12.2%) and animal interaction (11.1%)^53^. The current study included deaths that occurred within or external to a veterinary practice, which may in part account for why euthanasia risk was lower in these dogs (and hence risk of unassisted death higher) as many dogs may have died at the time of injury, during transport or on arrival to a veterinary practice or at home.

Dogs without a cause of death recorded had reduced odds of euthanasia (OR 0.08) compared with neoplasia. Since euthanasia must be performed by a veterinary surgeon, or by a person who has been authorised to do so by a veterinary surgeon^54^, it follows that euthanased dogs are more likely to have a disorder diagnosis prior to or on presentation for euthanasia. Bereaved pet owners report that they wanted to explore all possible treatment options before considering euthanasia^55^. Dogs that died unassisted may have died at home, at an emergency out of hours clinic or may not have visited a veterinary clinic for a specific problem prior to death and hence might not have a disorder diagnosis.

Complications associated with clinical care in dogs resulted in reduced odds of euthanasia (OR 0.08) compared with neoplasia. The prevalence, however, was relatively low with 0.2% dogs euthanased and 2.1% dogs dying unassisted due to complications. This highlights the value of considering both the absolute (prevalence) as well as the relative (odds) values when interpreting epidemiological results. “Complications” may have encompassed a range of specific factors, therefore future studies may help evaluate this finding further to determine if there are specific complications more likely to result in euthanasia or unassisted death and whether there are preventative measures that could be implemented, such as those designed to reduce postoperative complications in elderly human patients^56,57^.

Heart disease as a cause of death showed reduced odds of death by euthanasia (OR 0.14). There were 4.6% (1047) euthanasia cases attributed to heart disease compared with 19.7% (251) unassisted deaths. Cardiac disease is often subclinical, non-fatal or chronic^58^, therefore it may be that a proportion of the euthanased dogs died with heart disease (whether diagnosed or undiagnosed) rather than this being the definitive reason for euthanasia. Heart disease was the most common cause of unassisted death in dogs, highlighting management of this condition as important for veterinarians to discuss with owners of affected dogs. For example, one important area for such discussions may be the impact of breathlessness on quality of life; this clinical symptom is reported as having a profound impact on quality of life in human patients^59^.

After accounting for other confounding factors such as grouped-level disorder and age, Rottweilers were more likely to be euthanased (OR 1.76) than to die an unassisted death, compared with Labrador Retrievers, whilst seven breeds were more likely to die from unassisted deaths including Bulldog, Pug, Cavalier King Charles Spaniel and Yorkshire Terrier. The reasons for the breed risks identified are likely multifactorial, with possible differences including the risk of sudden death between breeds, that would preclude euthanasia. Heart disease has previously been identified as the most common cause of sudden and unexpected death in dogs^60^. In a recent VetCompass study, Yorkshire Terriers and Cavalier King Charles Spaniels had a significantly increased odds of degenerative mitral valve disease diagnosis compared with crossbred dogs^61^, a cardiac disorder where 50% of disorder-related deaths are considered ‘sudden’^62^.

Cause of death terms were taken in to account in the multivariable modelling, therefore the differences in breed risks identified may result from other factors that the model could not account for. Such factors might include owner characteristics e.g. attitudes towards pet death, and the dog-owner relationship. The finding that brachycephalic breeds including the Bulldog and Pug were at increased risk of unassisted death is of interest, given their current boom in popularity^63^. Although further research is needed, results of recent studies exploring the ownership behaviours and beliefs of owners of brachycephalic breeds may indicate that owning this breed type has the potential to influence when, and if, a dog is euthanased. Owners of brachycephalic breeds (specifically Bulldogs, Pugs and French Bulldogs) have been reported to form particularly strong dog-owner relationships^64^. However, the aforementioned study did not compare brachycephalic dogs to non-brachycephalic dogs, therefore the strength of the relationship cannot be quantified. Previous studies have found strength of the dog-owner bond is related to health-seeking behaviours in dog owners (e.g. owners with strong bonds seeking higher levels of veterinary care and being more likely to follow veterinary recommendations regardless of cost)^65^, research on brachycephalic dog owners reveals disparities in their perceptions of dog health versus those of veterinary professionals; these differences may disrupt tendency to follow veterinary advice that is shown by owners of dogs in general. Owners of brachycephalic breeds tend to ‘normalise’ poor health in their breed. Although they may be aware that their dog is showing signs of respiratory disease, for example, they may not consciously accept that this is a real problem for their dog, and instead attribute it as a ‘normal’ feature of their breed^66^. This phenomenon has been found to extend beyond wakeful respiratory dysfunction, and to also cover dysfunctional sleeping, thermoregulation and eating that are normalised in these breeds, such that dogs need to reach a critical level of clinical severity before owners consciously acknowledge their dog has a ‘problem’^64^. These normalisation and thresholding phenomena may affect euthanasia-decision making, whereby owners may fail to perceive their dog as ‘unwell’ and do not consider their dog’s quality of life as sufficiently impaired to justify euthanasia. Consequently, some severely affected dogs are more likely to die an unassisted death.

Bodyweight was a significant predictor of death by euthanasia, with dogs 20 to < 30 kg at 1.24 times the odds of euthanasia, relative to an unassisted death, compared with dogs < 10 kg. A previous study evaluating euthanasia or rehoming in dogs with behavioural issues reported an association between heavier bodyweight and increased risk of rehoming or euthanasia and/or the owners considering rehoming or euthanasia^67^. The reasons behind this association were not discussed in detail, however there is a disproportionate risk of injury associated with larger and/or more physically powerful breeds, as well as the existence of breed stereotypes, which may have contributed to this previous association^68^. Similarly, the precise reasons for differential euthanasia across weight categories in the current study are not clear. However, management considerations, such as the financial cost of treating larger dogs and difficulties in assisting with end of life care, might contribute to the association identified, although this has not been previously explored.

A greater proportion of euthanased dogs were communally cremated compared with dogs that died unassisted, whilst a greater proportion of dogs that died unassisted were individually cremated compared with euthanased dogs. In a previous study, Chur-Hansen et al. (2011) found that owners needed memorials, such as an animal’s ashes, to help them move through the grieving process, with some owners stating it felt as if their animal was not completely gone^69^. It is possible that owners whose dogs died unassisted may have desired to keep their dog’s remains (either through individual cremation or home burial) to aid in processing the grief of their loss. Conversely, owners of dogs who were euthanased may have benefited from a longer time processing the loss of their dog prior to their death, compared with those whose dog had an unassisted death, and therefore might not have felt as inclined to keep their dog in close proximity through home burial or retaining of ashes. Financial considerations may also play a part. Dogs that were euthanased may have been receiving prior treatment at a veterinary clinic, therefore owners may have opted for a less expensive method of managing remains following the cost of treatment. Conversely, dogs that died unassisted may not have been receiving costly treatment prior to death.

Decision-making about euthanasia involves the veterinarian and the animal owner(s). The veterinarian must shift focus from trying to cure the animal to admitting that there is nothing further that can be done, then convincing the client that the time has come for euthanasia^70^. Animal owners, in turn, appreciate the support of a veterinarian when making the decision for euthanasia in cases where treatment options have been exhausted^55^. The current study focuses on animal-related factors for euthanasia. However, client-related factors such as caregiver burden and financial constraints may lead to euthanasia decisions being made on grounds other than the animal’s quality of life and which could be the subject of future studies^71^.

This study has many similar limitations to previous studies based on retrospective primary-care data^2^. A large proportion of dogs that died unassisted did not have a cause of death reported (48.8%). Although a large proportion of these data were missing, dogs without a cause of death recorded were still included in the analysis to reduce bias. Some of the disorder groupings in the study might overlap, such as neoplasia and mass. The authors did not make any diagnostic assumptions i.e. disorders were recorded according to the attending veterinarian’s most specific diagnosis. Misclassification bias is possible in this instance, but may have been even greater if diagnoses are assumed. Euthanasia is a decision-making process involving owners, however the same cannot always be said for unassisted death and so this distinction should be accounted for when interpreting the results.

## Conclusions

The demographic and clinical factors associated with euthanasia and unassisted deaths were identified, which could be used by veterinarians to better understand the aspects influencing the euthanasia decision-making process in dogs. Poor quality of life and undesirable behaviour were major risk factors for euthanasia in dogs. Conversely, dogs with traumatic injury, complication associated with clinical care and heart disease showed increased risk of unassisted death. Older and heavier dogs also had higher odds of euthanasia. These findings could inform future studies, particularly qualitative research studies that might be designed to evaluate quality of life discussions, to develop measures to consider the appropriateness of palliative care for specific conditions and to assess the impact of euthanasia decision-making on owners and veterinary professionals.

## Methods

The study included all available dogs under primary veterinary care at clinics participating in the VetCompass Programme during 2016. Dogs under veterinary care were defined as those with either a) at least one electronic patient record (EPR) (VeNom diagnosis term, free-text clinical note, treatment or bodyweight) recorded during 2016 or b) at least one EPR recorded during both 2015 and 2017. VetCompass collates de-identified EPR data from primary-care veterinary practices in the UK for epidemiological research^29^. Data fields available to VetCompass researchers include a unique animal identifier along with species, breed, date of birth, sex, neuter status, insurance status and bodyweight, and also clinical information from free-form text clinical notes, summary diagnosis terms^72^ and treatment with relevant dates.

A cohort study design was used to estimate proportional euthanasia among deaths in dogs under primary veterinary care in the UK during 2016 and to report on demographic and clinical risk factors associated with euthanasia (relative to unassisted death). Sample size calculations in *Epi info (CDC)* estimated that approximately 185 dogs that were euthanased and 185 dogs that died unassisted would be required, to identify if dogs ≥ 6 years had at least twice the odds of euthanasia (relative to unassisted death) compared to dogs < 6 years, assuming 80% of dogs ≥ 6 years that die are euthanased, 80% power and 95% confidence^73^. Ethics approval was obtained from the RVC Ethics and Welfare Committee (reference number SR2018-1652).

Candidate death cases were identified using search terms appropriate to euthanasia and unassisted death in the clinical notes (euth*, pts*, crem* ashes, pento*, casket, beech, decease*, death, “put to sleep”, doa, died, killed, “home bury” ~ 1, “bury” and “home”) and in the treatment fields (euth*, pento*, crem*, casket, scatter, beech). The search findings were merged and a random selection of these was then manually reviewed in detail to identify dogs that died during 2016^74^. Death did not have to take place at the attending veterinary clinic, because VetCompass also captures information reported by owners to their veterinary clinics on deaths that occurred outside the clinic setting.

Method of death and cause of death were defined as two distinct entities. Method of death was categorized as euthanasia or unassisted, with missing data recorded as “Not recorded” and excluded from the statistical analysis. Cause of death described the main or first reported biomedical cause for the death regardless of the method of death. Cause of death terms were mapped to a grouped-level of diagnostic precision. Grouped-level terms mapped the original diagnosis terms to a general level of diagnostic precision (e.g. inflammatory bowel disease would map to gastro-intestinal) as previously described in the literature^74^. In order to maintain sufficient power for analysis, a combined disorder list from all of the 15 most common grouped disorders from dogs that were euthanased and dogs that died unassisted was generated and compared in the analysis. Dogs without a specific cause of death recorded were categorised and included in the analyses as “Not recorded”. In addition, method of dealing with the remains was reported and categorised as “Communal cremation”, “Individual cremation”, “Burial”, “Other” or “Not recorded”.

Breed information entered by the participating practices was cleaned and mapped to a VetCompass breed list derived and extended from the VeNom Coding breed list (The VeNom Coding Group, 2019). In order to maintain sufficient power for analysis, the breed variable included specific breeds with at least 20 dogs in either the euthanasia or unassisted death grouping respectively. Remaining dogs were grouped in to “Purebred—Other” and “Crossbred”. Neuter status was defined by the final available EPR neuter value and was combined with sex to create four categories: female entire, female neutered, male entire and male neutered. Bodyweight was defined as the bodyweight (kg) value closest to the date of death for each dog. Bodyweight (kg) was categorised: < 10, 10 to < 20, 20 to < 30 and ≥ 30. Age (years) was defined at the date of death and was categorised: < 6.0, 6.0 to < 9.0, 9.0 to < 12.0, 12.0 to < 15.0 and ≥ 15.0. Veterinary group attended was categorised as 1–5, based on 5 practice groups involved in the study. Insurance status was categorised as insured or not insured. Missing data were recorded as “Not recorded” and included in the analysis if this category accounted for > 10% of the study variable. Following data checking for internal validity and cleaning in Excel (Microsoft Office Excel 2013, Microsoft Corp.), analyses were conducted using SPSS version 24.0 (IBM Corp).

The proportion of deaths by euthanasia and unassisted were reported. All continuous variables were non-normally distributed and so were summarised using median, interquartile range (IQR) and range. Mann–Whitney U test, chi-square test and Fisher’s exact test were used as appropriate for comparison of demographic data between euthanasia cases and unassisted deaths^75,76^. Binary logistic regression modelling was used to evaluate univariable associations between risk factors (*grouped-level disorder, breed, bodyweight, age, sex-neuter* and *insurance*) and outcome. Outcome was defined as either euthanasia or unassisted death. Dogs with missing data on the method of death were recorded as “Not recorded” and excluded from further analysis. Euthanasia was the event of primary interest, therefore the model reported the odds of euthanasia relative to unassisted death for each category within a variable. Neoplasia was used as the baseline in the grouped-level disorder variable, since neoplasia has been reported as the most common cause of death in dogs under primary veterinary care^2^. Labrador Retriever was used as the baseline in the breed variable, rather than crossbreed which has been common in previous studies^77^, since Labrador Retrievers are more standardised with regard to genetics, bodyweight, skull shape and conformation compared to crossbreeds^78^.

Risk factors with liberal associations in univariable modelling (*P* < 0.2) were taken forward for multivariable evaluation. Model development used manual backwards stepwise elimination. Vet Group attended was evaluated as a fixed effect. Potential confounders were assessed by checking for a marked change in the odds ratio (OR) after removal of the variable from the model. Collinearity was investigated by examining the variance inflation factor (VIF) and tolerance, with collinearity indicated if VIF > 10 and tolerance < 0.1^79,80^. The area under the ROC curve and the Hosmer–Lemeshow test were used to evaluate the quality of the model fit. Statistical significance was set at the 5% level.

### Ethics approval

Ethics approval was granted by the RVC Ethics and Welfare Committee (reference number URN Ref SR2018-1652).

### Consent for publication

Not applicable.

## Data Availability

The datasets generated during and/or analysed during the current study are available at the RVC Research Online repository: https://researchonline.rvc.ac.uk/id/eprint/13486.

## Abbreviations

CI: Confidence interval
EPR: Electronic patient record
IQR: Interquartile range
KC: The Kennel Club
OR: Odds ratio
QOL: Quality of Life
